# Magnesium sulphate therapy in eclampsia: the Sokoto (ultra short) regimen

**DOI:** 10.1186/1756-0500-2-165

**Published:** 2009-08-19

**Authors:** Bissallah A Ekele, Danjuma Muhammed, Lawal N Bello, Ibrahim M Namadina

**Affiliations:** 1 Department of Obstetrics and Gynecology, Usmanu Danfodiyo University Teaching Hospital, Sokoto, Nigeria; 2 Department of Obstetrics and Gynecology, Specialist Hospital, Sokoto, Nigeria

## Abstract

**Background:**

Continuing the administration of magnesium sulphate for 24 hours after the last fit in patients with eclampsia is at best empirical. The challenge of such a regimen is enormous in low-resource countries. The objective of this study was to assess the effectiveness of an ultra-short regimen of magnesium sulphate in eclamptics.

**Findings:**

This was a prospective, cohort study of eclamptic patients admitted between July 2007 and June 2008 that were given 4 grams magnesium sulphate intravenously and 10 grams intramuscularly (5 grams in each buttock) as the sole anticonvulsant agent. Main outcome measure was the absence of a repeat fit. Other aspects of eclampsia management were as in standard practice. One hundred and twenty one (121) patients were managed with this regimen. There were 29 ante partum, 76 intrapartum and 16 post partum cases of eclampsia. Most of the patients were primigravidae (100; 83%) with an average age of 18.7 years. There were nine cases (7.4%) of recurrent fits that occurred within four hours of the loading dose. One recurrent fit occurred in the ante partum group, seven in the intra partum and one in the post partum group. There were 12 maternal deaths giving a case fatality rate of 9.9%.

**Conclusion:**

Limiting the dosage of magnesium sulphate to 14 grams loading dose (4 grams intravenous and 10 grams intramuscular) was effective in controlling fits in 92.6% of cases in the study group. A properly conducted, randomized controlled trial is needed to test our proposed regimen.

## Background

Pre-eclampsia and eclampsia account for about 9% of maternal deaths in Africa and Asia and about one-quarter of maternal deaths in Latin America and the Caribbean [1]. In some parts of northern Nigeria, eclampsia alone contributes to almost one third of maternal mortality [2-4]. It is has been established that magnesium sulphate is the anticonvulsant of choice for both prevention and treatment of eclampsia [5,6]. But the routine 24-hour administration of maintenance doses of magnesium sulphate after a loading dose to all patients with eclampsia [7,8] has not been properly subjected to scientific scrutiny.

The exact mechanism of action of magnesium sulphate is also not clearly understood. Some workers have suggested the blockade of N-methyl D-aspartate (NMDA) receptors involved in seizure genesis [9] or calcium channel blocking, preventing cerebral vasospasm [10]. The concept of 'one-size-fits-all' in magnesium sulphate administration is also as curious as it is interesting.

A potential concern for magnesium sulphate therapy is the risk of side effects which could increase with the duration of treatment especially if there are challenges in clinical monitoring of the patients. Similarly, the cost of therapy would inevitably increase with the duration of treatment. For example with the standard Pritchard regimen [8] in which 5 grams of magnesium sulphate is administered four-hourly for 24 hours after loading with 14 grams; a patient would require at least 44 grams of the drug to complete a course. And at the retail pharmacy where 10 mls ampoule of 50% magnesium sulphate cost one hundred and fifty naira (about a dollar), a patient would require one thousand two hundred naira (about nine dollars) for a course of the drug. Meanwhile, only one-third of the drug (14 grams) and the amount (about three dollars) would have been required if the regimen was limited to just the loading dose. Although the drug is portrayed as cheap in most western literature and the developed countries, it is neither cheap nor easily available in many developing countries [11]. Therefore any effective regimen that would require less medication and labor would be attractive to health care providers especially in resource-poor countries. Begum and co-workers [12] had earlier reported the effectiveness of a low dose regimen for eclamptics in which the loading dose was reduced from the usual 14 grams to 10 grams and 2.5 grams (instead of the traditional 5 grams) was administered four-hourly for 24 hours as maintenance doses. Ekele and Ahmed [13] previously reported success with a 12-hour maintenance regimen instead of the traditional 24 hours and there was a recent report from India with smaller doses [14]. The question still being asked is whether the loading dose alone would suffice [15]? Incidentally most of the quest for smaller, shorter or simpler but effective and safe regimen seems to be coming from researchers in developing countries probably because of the peculiar challenges and constraints in those settings.

The objective of this study was to determine the effectiveness of an ultra short regimen of magnesium sulphate in the control of fits in patients with eclampsia.

## Methods

This was a prospective, cohort study of patients with eclampsia managed from July 2007 to June 2008 at the Maternity Unit, Specialist Hospital, Sokoto, Nigeria, after obtaining institutional ethical approval.

Sokoto, where the study was undertaken is the capital city of Sokoto state in north western Nigeria. Islam is the predominant religion of the people with the practice of *'purdah' *(women seclusion) and early girl marriage is not uncommon in the community.

The hospital is a state-owned tertiary health facility located in the centre of the town. Apart from attending to referred cases, it has liberal admission policy including the acceptance of patients that come directly from their homes for normal delivery and those with complications.

Eclampsia is diagnosed if there was history of generalized tonic-clonic convulsions, elevated blood pressure and proteinuria provided there was no previous history of seizure disorders like epilepsy. If there was doubt about the diagnosis because the accompanying relation(s) did not witness the seizure and there was no elevated blood pressure at admission, the patient was excluded from the study. Also excluded from the study were six patients that had diazepam as anticonvulsant prior to admission. At admission each patient that fulfilled the inclusion criteria had 4 gm of magnesium sulphate administered slowly intravenously over 10 minutes and 5 gm given deep intramuscularly in each buttock making a total loading dose of 14 gm. If there was a repeat fit after the loading dose, an additional 2 gm magnesium sulphate was given intravenously and the maintenance dose of 5 gm intramuscularly was then continued for 12 hours of delivery or the last fit depending on which occurred later. Hydralazine injection was the antihypertensive used to control severe hypertension and was administered as intermittent bolus doses to keep diastolic blood pressure at about 90 mmHg. Methyldopa and nifedipine were the long term anti-hypertensive agents used. Obstetric management was carried out after stabilizing each patient. Patients with unfavorable cervix but adequate pelvis had induction of labor after cervical ripening with Misoprostol. Those admitted in labor with no contraindication for vaginal delivery were monitored and allowed vaginal delivery. Cesarean section was indicated for cephalo-pelvic disproportion or fetal distress. Craniotomy was performed if the patient was already in obstructed labor on admission with fetal demise. Babies with birth asphyxia and low birth weights were managed at the Special Care Baby Unit.

## Results

One hundred and twenty one patients (121) were managed using this regimen. There were 2,313 total deliveries within the study period given an incidence of 5.2% of maternities. Most of the patients (96 cases; 79%) had no antenatal care. The age range was from 14 years to 38 years with a mean of 21 years. There were 100 primigravidae (83%) of a mean age of 18.7 years. The eclamptic type was 29 ante partum (had eclampsia but not in labor on admission), 76 intra partum (labor pains predated the convulsion) and 16 post partum cases. Most (58) of the intra-partum type of eclampsia came in advanced stage of labor with no fetal heart activity and vaginal delivery was achieved in those cases. There were 7 cases of craniotomy for obstructed labor with fetal demise and 14 patients had caesarean sections (11.5%) for cephalo-pelvic disproportion or fetal distress.

Repeat fits occurred in 9 patients (one ante partum, seven intrapartum and one post partum). None of those that had craniotomy had repeat fit. In 6 of the patients the fits reoccurred within the first hour of loading dose while in the remaining 3 the fits reoccurred within 4 hours of the loading dose. This was the group that had the 12-hour maintenance doses after aborting the repeat fit with an additional 2 grams of magnesium sulphate.

There were 12 maternal deaths (7 from cardiopulmonary failure, 4 from cerebro-vascular accident and one from septicemia following caesarean delivery for obstructed labor with live fetus). Only one of the maternal mortalities was in the recurrent fit group and cerebro-vascular accident was the probable cause of death. Case fatality rate was 9.9%. There were 67 still births (58 of the fetal deaths occurred before hospital admission).

## Discussion

The incidence of eclampsia within the study period was 5.2% which is similar to figures obtained from some centers in Nigeria [3,4,16] and underscores the magnitude of the disease even though it could be argued that the calculated rate was based on number of hospital deliveries rather than being population-based. Other factors found associated with eclampsia in this study were young maternal age; primigravidity and lack of prenatal care which are similar to what have been reported by other workers [16,17].

The ultra short regimen of 14 grams loading dose of magnesium sulphate was effective in 92% of cases in this study. The concept of using just the loading dose of magnesium sulphate to control and prevent fits in eclampsia was suggested by Boyd and Brower [18] but the first comprehensive study was published in 2002 [19]. It was shown in that study that the recurrent convulsion rate was almost the same (3.96% versus 3.52%) between the group that had only a loading dose of 10 grams (4 gram intravenous and 6 gram intramuscular) and the control group that had both the loading dose and the maintenance doses of 2.5 grams four-hourly for 24 hours. However, one of the limitations of the study was that it was not double-blind with the possibility of allotment bias. The main difference between our study and the loading dose arm of that study was that we used 14 grams but they used 10 grams and we recorded a 7.4% recurrent fit as against their 3.9%.

Why would small doses or an ultra short regimen of magnesium sulphate be effective in eclamptics? We do not have an explanation but some workers have suggested a small body mass index [19]. Unfortunately, it was very difficult to weigh and calculate body mass index of most of the patients in our study for they were either deeply unconscious or in advanced stage of labor at the time of admission. Even then a small body mass index alone might not explain the whole phenomenon, for some authors have shown that there was no association between treatment failures and body mass or with serum magnesium levels [20].

The ultra short regimen used in this study is attractive for many reasons. In the first place, it minimizes the risk of toxicity since most patients would not need the maintenance doses. Clinical monitoring for magnesium sulphate toxicity as prescribed by eliciting patellar reflexes, counting respiratory rate and measuring urinary output could be religiously undertaken at tertiary hospitals where there are adequate manpower, facilities and supervision. But manpower in particular is a challenge at the district hospitals and primary health care centers of many developing countries. We have also observed that in some patients with eclampsia, the deep tendon reflexes could be adjudged equivocal or absent even before the initiation of magnesium sulphate therapy if there is a decision to administer the drug; hence tendon reflexes cannot be completely relied upon as an early sign of toxicity under such a situation. Secondly, the cost of treatment using the ultra short regimen is about a third of the standard regimen and this would be a welcome development in resource poor countries. Perhaps of immense importance is the observation from this study that majority of eclamptic patients that would have a repeat fit will do so within one hour of the loading dose and thereby giving the health worker the opportunity to identify those that would need the maintenance doses.

A case fatality rate of 9.9% even though high is still better than the 29% recently reported from Nigeria [21]. Larger trials have found that magnesium sulphate when used as anticonvulsant for eclampsia is associated with low maternal deaths [6,19,22].

The very poor perinatal outcome in this study is of serious concern and very disturbing but there were as many as 58 fetal deaths at the time of admission of the patients. It is sequel to late presentation to the health facility. Many reasons have been advanced for eclamptic patients presenting late amongst which is the belief that eclampsia is caused by '*iska*' which translate literarily to mean 'spirit' thus treatment from orthodox medicine is sought only after other options have failed. Also it might take time to get the necessary clearance and permission from the husband for a woman to be allowed out of the house even at times of emergency especially if the man is not at home. This is the subject of an on-going research (Adamu AN; *personal communication*).

## Conclusion

In conclusion, the ultra-short protocol of 14 grams magnesium sulphate given 4 grams intravenously and 10 grams intramuscularly was effective as an anticonvulsant in 92.6% of eclampsia patients in the study area. Only 7% of the patients needed the continuation of the maintenance doses. However a properly conducted, multi-center, randomized controlled trial is recommended to test this proposed regimen.

## Competing interests

The authors declare that they have no competing interests.

## Authors' contributions

BAE conceived the study and participated in the design and manuscript writing; DM was part of the study design, administered most of the drug and was part of manuscript writing, LNB was part of the study conception and participated in manuscript writing while IMN contributed to the study design and the draft manuscript. All authors read and approved the final manuscript.
